# MRI-Based Assessment of Risk for Stroke in Moyamoya Angiopathy (MARS-MMA): An MRI-Based Scoring System for the Severity of Moyamoya Angiopathy

**DOI:** 10.3390/diagnostics14131437

**Published:** 2024-07-05

**Authors:** Leonie Zerweck, Constantin Roder, Ganna Blazhenets, Peter Martus, Johannes Thurow, Patrick Haas, Arne Estler, Georg Gohla, Christer Ruff, Nadja Selo, Urs Würtemberger, Nadia Khan, Uwe Klose, Ulrike Ernemann, Philipp T. Meyer, Till-Karsten Hauser

**Affiliations:** 1 Department of Diagnostic and Interventional Neuroradiology, University Hospital Tuebingen, 72076 Tuebingen, Germanytill-karsten.hauser@med.uni-tuebingen.de (T.-K.H.); 2 Department of Neurosurgery, University Hospital Tuebingen, 72076 Tuebingen, Germany; constantin.roder@med.uni-tuebingen.de (C.R.); patrick.haas@med.uni-tuebingen.de (P.H.);; 3 Department of Nuclear Medicine, Medical Center, Medical Faculty, University of Freiburg, 79106 Freiburg im Breisgau, Germany; 4 Institute for Clinical Epidemiology and Applied Biometry, University Hospital Tuebingen, 72076 Tuebingen, Germany; 5 Department of Neuroradiology, Medical Center, Faculty of Medicine, University of Freiburg, 79106 Freiburg im Breisgau, Germany; 6 Moyamoya Center, University Children’s Hospital Zurich, 8032 Zurich, Switzerland

**Keywords:** moyamoya angiopathy, scoring system, MRI, [^15^O]water PET, cerebral perfusion reserve capacity

## Abstract

Before revascularization, moyamoya patients require hemodynamic evaluation. In this study, we evaluated the scoring system *Prior Infarcts*, *Reactivity and Angiography in Moyamoya Disease* (PIRAMID). We also devised a new scoring system, *MRI-Based Assessment of Risk for Stroke in Moyamoya Angiopathy* (MARS-MMA), and compared the scoring systems with respect to the capability to predict impaired [^15^O]water PET cerebral perfusion reserve capacity (CPR). We evaluated 69 MRI, 69 DSA and 38 [^15^O]water PET data sets. The PIRAMID system was validated by ROC curve analysis with neurological symptomatology as a dependent variable. The components of the MARS-MMA system and their weightings were determined by binary logistic regression analysis. The comparison of PIRAMID and MARS-MMA was performed by ROC curve analysis. The PIRAMID score correlated well with the symptomatology (AUC = 0.784). The MARS-MMA system, including impaired breath-hold-fMRI, the presence of the Ivy sign and arterial wall contrast enhancement, correlated slightly better with CPR impairment than the PIRAMID system (AUC = 0.859 vs. 0.827, Akaike information criterion 140 vs. 146). For simplified clinical use, we determined three MARS-MMA grades without loss of diagnostic performance (AUC = 0.855). The entirely MRI-based MARS-MMA scoring system might be a promising tool to predict the risk of stroke.

## 1. Introduction

Moyamoya angiopathy (MMA) is a progressive steno-occlusive cerebrovascular disease that predominantly affects the terminal parts of the internal carotid artery (ICA), as well as the proximal branches of the middle cerebral artery (MCA) and the anterior cerebral artery (ACA) [1,2,3]. Patients with MMA typically present with transient ischemic attacks (TIA), ischemic or hemorrhagic strokes and headache [2,3,4,5].

The diagnosis of MMA is based on conventional angiographic imaging [3], where reactively formed collateral vessels surrounding the stenoses appear as a “puff of smoke” (Japanese: “moyamoya”), which gave the disease its name [2,6]. Further diagnostic evaluation usually includes different sequences of structural MRI, where ischemic or hemorrhagic lesions can be detected, and MRI-based angiography [6]. The latest studies also propose MRI-based high-resolution arterial vessel wall imaging to identify patients with disease progression [6,7]. The indication for surgical revascularization to prevent strokes is mainly based on the evaluation of the cerebral perfusion reserve capacity (CPR) [8,9,10,11,12], which is intended to provide information about the risk of ischemic stroke [13]. The nuclear medicine method [^15^O]water PET with acetazolamide (ACZ) challenge is the gold standard for the estimation of the CPR [14]. Hypercapnia-triggered BOLD MRI has been used increasingly to evaluate the cerebrovascular reactivity (CVR), which is considered as a surrogate marker of the CPR [8,10,14,15,16].

Recently, Ladner et al. outlined a multimodal scoring system to classify the severity of MMA, titled *Prior Infarcts*, *Reactivity and Angiography in Moyamoya Disease* (PIRAMD), and proposed it for clinical decision-making and for the evaluation of an intervention response [17]. Van Niftrik et al. validated the scoring system in 38 patients with MMA [18]. The PIRAMID scoring system is based on digital subtraction angiography (DSA), structural MRI and hypercapnia-triggered BOLD MRI and correlates well with the past and current neurological symptomatology [17].

The first aim of this study was to evaluate the PIRAMID scoring system in a large number of patients by applying the scoring system to neuroradiological data sets (DSA, structural and functional MRI) and by investigating the capability to predict past and current neurological symptomatology as proposed by Ladner et al. We aimed to evaluate the scoring system not only in the ICA territories but also specifically in the ACA and MCA territories separately.

In our opinion, the indication for revascularization surgery should not mainly be based on patients’ past or current neurological symptoms, as the goal is to prevent ischemic events beforehand. Specifically, in (currently) asymptomatic patients, the aim is to avoid ischemic strokes/neurological symptoms. Therefore, therapeutic considerations should rather be based on the evidence of a decreased CPR indicated by [^15^O]water PET, which plays an important role in clinical decision-making in patients with MMA [9,10,14], including those without ischemic symptoms.

Hence, as the second aim of this study, we intended to validate the PIRAMID scoring system regarding the capability to predict a reduced CPR. In this context, our aim was to investigate whether a classification system with a stronger ability to predict an impaired CPR on [^15^O]water PET in the ACA and MCA territories can be established with multimodal neuroradiological imaging. Here, we propose an entirely MRI-based scoring system called *MRI-Based Assessment of Risk of Stroke in Moyamoya Angiopathy* (MARS-MMA).

## 2. Materials and Methods

### 2.1. Patient Selection

A retrospective analysis of MRI, DSA and [^15^O]water PET data sets and clinical examinations of patients with MMA was performed. All imaging scans were performed as routine clinical scans between 2018 and 2023. The inclusion criteria of the study were angiographically proven MMA and the availability of MRI, DSA and a clinical examination at most 120 days apart, with no revascularization therapy in between. Only data sets of patients without prior bilateral surgical revascularization were used. In the case of unilateral revascularization, the data of the non-revascularized hemispheres were included in the study. If available, [^15^O]water PET data sets were included in the study. The exclusion criterion was a secondary cerebral disease. Ethical approval was obtained from the local ethics committee.

### 2.2. Neurological Assessment

All territories were analyzed with respect to past or current neurological symptomatology, which was retrospectively derived from the electronic medical record. Analogous to Ladner et al., neurological symptomatology was defined as either a history of recurrent TIA or persistent motor, sensory or language deficits referable to a vascular territory, while non-referable psychological symptoms or headache were not included [17].

### 2.3. MRI Data Acquisition and Evaluation

**Figure 1:**
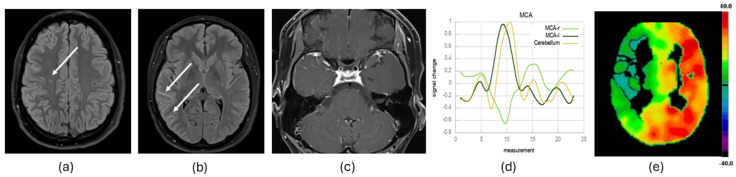
Exemplary MRI and [^15^O]water PET data evaluation of one patient. Each territory of the anterior cerebral artery and the middle cerebral artery (MCA) was evaluated with respect to postischemic FLAIR lesions > 4 mm ((**a**), see arrow), Ivy sign on axial FLAIR images ((**b**), see arrows), arterial vessel wall contrast enhancement (**c**) and >50% reduced breath-hold fMRI cerebrovascular reactivity (bh-fMRI CVR) response compared to the cerebellar bh-fMRI CVR response ((**d**), see the right MCA territory (MCA-r)), as well as at least a severely reduced cerebral perfusion reserve capacity after acetazolamide administration ((**e**), see right MCA territory).

All MR images were acquired on a 3 T MR Scanner (Magnetom Skyra, Siemens, Erlangen, Germany) using a standard 20-channel head coil. At our Moyamoya center, we use a standardized protocol of MR imaging, including, among others, FLAIR/T2-weighted sequences, a post-contrast fat-saturated T1 black blood sequence and a T2*-weighted echo-planar imaging (EPI) sequence (see Figure 1), as described in more detail in the following.

#### 2.3.1. Structural MRI Data Evaluation

The quantification of ischemic lesions in MRI was performed by analyzing the FLAIR and/or T2-weighted sequences. As proposed by Ladner et al., a size criterion of a minimum axial diameter of 4 mm was used to separate lacunar infarcts from white matter changes [17]. The vascular territory of the ICA, as well as the territories of the ACA and the MCA, were rated in consensus by two neuroradiologists.

#### 2.3.2. Functional MRI Data Acquisition, Processing and Evaluation

The bh-fMRI data were measured by means of T2*-weighted echo-planar imaging (EPI) sequences with the following parameters: TR = 3000 ms, TE = 36 ms, matrix 96 × 96, 3 mm slice thickness, 34 slices in interleaved ascending order, FOV = 245 mm, resolution 2.6 × 2.6 × 3.0 mm, echo spacing 0.58 ms, TA 6:53 min, 135 measurements.

The breath-hold task consisted of 60 s of regular breathing, followed by five repetitive cycles, each consisting of 9 s end-expiratory breath-hold periods and 60 s of normal breathing. The respiratory instructions were presented visually by means of a wall-mounted display and a mirror fixed to the head coil. Presentation V20.1 (Neurobehavioral Systems, Berkeley, CA, USA) was used to present the scanner-triggered stimuli.

The MRI data preprocessing was performed in Statistical Parameter Mapping (SPM12) (https://www.Fil.Ion.Ucl.Ac.Uk/spm/ URL (accessed on 10 June 2024) running on MATLAB (R2018b (The MathWorks, Inc., Natick, MA, USA; http://www.mathworks.com)). The DICOM images were converted to NifTI slice timing corrected to equalize the time of image acquisition, realigned to correct for patients’ head movement, normalized to the standard MNI space and segmented with the use of binary masks (vascular territories of the ACA, MCA and cerebellum) [19,20]. Further processing of the data was performed by using in-house scripts programmed in MATLAB. As the cerebellum represents a region where a physiological hemodynamic response is expected in patients with MMA [10,15,21], the mean cerebellar signal time course of each of the five repetitive cycles was calculated to verify patients’ compliance in performing the breath-hold task. In the case of the incorrect execution of the breath-hold paradigm, individual cycles could be excluded from further evaluation [10,15,21,22]. For a more detailed description, we refer to the study of Hauser et al. [10]. Next, the signal time courses of all included breath-hold cycles were averaged and the VOI mean signal time course of the above-mentioned masks was calculated. The relative BOLD signal change was calculated to adjust for variability in the baseline signal intensity of the EPI sequence. The relative reduction [%] (in steps of 10% from 0% to 100% and steal phenomenon) of the BOLD signal peak in comparison to the cerebellar BOLD signal peak was analyzed in the ACA and the MCA territories (see Figure 1). The value with the lower CVR was considered the CVR value for the respective ICA territory.

#### 2.3.3. Arterial Wall Contrast Enhancement Evaluation

Arterial vessel wall contrast enhancement was analyzed separately in the C1–C7 segments, the M1 and M2 segments and the A1 and A2 segments of each hemisphere and the anterior communicating artery by a neuroradiologist with extensive experience in neurovascular imaging. We distinguished between mild, moderate and strong contrast enhancement as proposed by Roder et al. [7]. Mild enhancement was defined as an only faint band of hyperintensity, moderate enhancement was defined as a clear band of hyperintensity with a lower signal intensity than in the pituitary stalk and strong enhancement had a signal intensity of at least the pituitary stalk [7] (see Figure 1).

To simplify the rating of the evaluated vascular territories (ACA and MCA), we considered the ACA and MCA as affected if one segment of the ACA/MCA or the supplying ICA showed a vessel wall contrast enhancement. We distinguished between mild, moderate and strong contrast enhancement and classified each vascular territory according to the segment with the strongest contrast enhancement.

#### 2.3.4. Ivy Sign Evaluation

Prominent leptomeningeal collaterals along the cortical sulci and the subarachnoid space with slow blood flow yield strong signals on FLAIR images, resulting in a radiographic finding called the Ivy sign [23,24] (see Figure 1). Each evaluated vascular territory (territories of the ACA and the MCA) was binarized in consensus by two neuroradiologists based on the presence or absence of the Ivy sign.

### 2.4. Digital Subtraction Angiography Data Acquisition and Evaluation

All DSA (6-vessel catheter angiogram) data were acquired during routine clinical examinations. All angiographic rating was performed by a neuroradiologist with extensive experience in neurovascular interventions.

Similarly to Ladner et al., the modified Suzuki Score (mSS) was used to account for stenoses of the ICA, the MCA and the ACA and the presence or absence of lenticulostriate collaterals [17]. The mSS ranges from 0 to IV, with more severe disease represented by higher values [17]. Analogously to Ladner et al., for simplification, we considered the mSS impaired if it accounted for ≥II [17].

Additionally, analogously to Ladner et al., regional collateralization was evaluated by dividing the DSA into 7 anatomical sites based on the ASPECTS-defined regional vascular territories (M1–M6 and basal ganglia) [17]. A vascular territory was defined as impaired if no or only peripheral collaterals supplying the ischemic site were visible [17]. A territory was considered not impaired if there was a normal anterograde flow or if the ischemic bed was completely irrigated by a collateral flow [17]. Analogously to Ladner et al., the total number of impaired territories (0–7) was counted for each MCA territory, and, for the simplification of the scoring system, the collateralization of the MCA was considered impaired if ≥2 territories were impaired [17].

Since we intended to evaluate not only the ICA territories but also the MCA and ACA territories separately, we constructed a rating of the ACA territory, analogous to the one proposed above, namely the ASPECTS-based rating of the MCA. The arterial territories atlas [25] was adapted to divide the ACA territories further into the anterior part (orbitofrontal, frontopolar, anterior and middle frontal arteries) and posterior part (posterior frontal, precentral, superior and inferior parietal branches).

### 2.5. [^15^O]Water PET Data Acquisition and Evaluation

PET scans were acquired as part of the clinical routine MMA evaluation on a Philips Gemini (*n* = 3) TF64 or Vereos (*n* = 35) digital PET/CT system and analyzed as previously described [10,15]. In brief, two 4 min PET scans each (bolus injection of 300 MBq [^15^O]water per scan) were performed before (baseline) and after a 5 min infusion (10 mL) of a standard dose of 1000 mg ACZ (post-ACZ). The inter-scan interval was 10 min, with the ACZ infusion being started immediately after the second baseline scan (i.e., the first post-ACZ scan started 10 min after start of the ACZ infusion). CPR maps were calculated as the voxel-wise signal change [%] between the averaged baseline and post-ACZ maps (in analogy to [26]), which were derived by integrating dynamic PET images over 60 s after the arrival of the tracer in the individual patient’s brain [27].

Each individual’s CRP map was rated by two experienced, board-certified nuclear medicine specialists in consensus in a highly standardized manner. Maps were displayed in 32 transaxial planes covering the entire brain using a harmonized “rainbow” color scale. The color scale was thresholded symmetrically around zero (i.e., max and min set to ± [mean value + 2 standard deviations of CPR in cerebellar and PCA territories]) and customized such that the zero transition (i.e., watershed in the case of CBF steal) was marked by three black bins within the 256-bin color-scale. The ACA, MCA and PCA territories (separately for both hemispheres) were rated for CRP using a 4-point score: normal/mildly reduced CPR (1), moderately (2) and severely (3) reduced CPR or steal phenomenon (negative CPR) (4). Normal/mild, moderate and severe corresponded approximately to the upper, middle and lower thirds of the positive CPR windowing. For simplicity, in the following, we define “severe CPR impairment on [^15^O]water PET” as a visual CPR score of ≥3 (i.e., severely reduced or steal phenomenon) (see Figure 1). We binarized each ACA/MCA territory regarding a severe CPR impairment or a normal/not severely impaired CPR.

### 2.6. Validation of the PIRAMID Scoring System

All statistical analyses were performed using SPSS Statistics (IBM Corp. Released 2021. IBM SPSS Statistics for Windows, Version 28.0. IBM Corp: Armonk, NY, USA).

Before we calculated the PIRAMID scores of each territory, analogously to Ladner et al., we performed a preliminary analysis to investigate the percentage of BOLD signal reduction that predicted a severe CPR impairment with the highest sensitivity and specificity. For this purpose, we performed a receiver operating characteristic (ROC) curve analysis using [^15^O]water PET as the dependent variable and the relative BOLD signal reduction as the independent variable. The ROC analysis was performed for the ICA and the ACA/MCA territories separately. The relative BOLD signal reduction with the maximum Youden index was used as a threshold to binarize the CVR of each evaluated territory as severely impaired or not.

Next, we calculated the PIRAMID scores of each patient’s ICA territory, but also of each ACA and MCA territory, by weighting the binary components, as proposed by Ladner et al. [17]. For the presence of a prior infarct, one point was given, and, for the presence of an impaired CVR, a mSS ≥ II and the presence of ≥2 territories with impaired collaterals, three points were given, resulting in a total of up to ten points. The territories were divided into three PIRAMID grades based on the PIRAMD score as follows: Grade 1, 0–5 points; Grade 2, 6–9 points; and Grade 3, 10 points [17].

To validate the PIRAMID scoring system, we performed an ROC curve analysis using neurological symptomatology as the dependent variable and the PIRAMID score, the PIRAMID grade and all components of the PIRAMID scoring system (prior infarcts, impaired CVR, mSS ≥ II, ≥2 territories with impaired collaterals) as the independent variables.

The validation of the PIRAMID score and grade was performed for two regions (ICA territory of each hemisphere) and four regions (MCA and ACA territories of each hemisphere) separately.

### 2.7. Development and Evaluation of the MARS-MMA Scoring System

Our second aim was to investigate whether another scoring system might predict the severe CPR impairment of the ACA and MCA territories better than the PIRAMID scoring system. For this purpose, we propose an optimized new scoring system incorporating the components of the PIRAMID scoring system and additionally the presence of arterial vessel wall contrast enhancement and the Ivy sign on FLAIR images.

First, we calculated, in a preliminary analysis, the grade of arterial wall contrast enhancement indicating a severe [^15^O]water PET impairment with the highest sensitivity and specificity. For this purpose, we performed an ROC curve analysis using [^15^O]water PET as the dependent variable and the arterial vessel wall contrast enhancement as the independent variable. The grade of contrast enhancement with the maximum Youden index was determined as the optimal cut-off to binarize the CVR of each evaluated territory in the further analysis as impaired or not impaired.

Afterwards, we performed a backward and forward binary logistic regression analysis with [^15^O]water PET CPR impairment as the dependent variable and all components of the PIRAMID scoring system (prior infarcts, impaired CVR, mSS ≥ II, ≥2 territories with impaired collaterals), as well as the presence of an arterial wall contrast enhancement and the presence of the Ivy sign, as the independent variables. A new scoring system (MARS-MMA) was created with all factors, revealing the significant prediction of a severe [^15^O]water PET CPR impairment at *p* < 0.05. The relative weighting for each factor was determined by the calculated regression coefficients of the binary logistic regression analysis.

For clinical use, the MARS-MMA scoring system was divided into three grades, for which two threshold values had to be defined. After the determination of the components of the MARS-MMA score and their weightings, all possible MARS-MMA scores that the territories could achieve by combining the weighted factors were calculated. Grade stratification was performed by calculating the individual grades of each ACA/MCA territory for all possible pairs of threshold values. The optimum pair of threshold values was calculated with ROC curve analysis using [^15^O]water PET CPR impairment as the dependent variable and the grades of all possible pairs of threshold values as the independent variables.

The MARS-MMA score and grade were calculated for each ACA/MCA territory. As our aim was to compare the newly developed MARS-MMA scoring system to the PIRAMID scoring system, we calculated a so-called *adapted PIRAMID score* to avoid the asymmetric overfitting of the new MARS-MMA scoring system. For this purpose, we performed a binary logistic regression analysis with [^15^O]water PET CPR impairment as the dependent variable and all components of the PIRAMID scoring system (prior infarcts, impaired CVR, mSS ≥ II, ≥2 territories with impaired collaterals) as the independent variables and optimized the factors’ weights based on the regression coefficients to fit our data.

The comparison between the MARS-MMA scoring system and the PIRAMID scoring system was conducted by performing an ROC curve analysis with [^15^O]water PET CPR impairment as the dependent variable and the MARS-MMA score and the adapted PIRAMID score as independent variables. The Akaike information criterion (AIC) was calculated to account for different numbers of predictors.

## 3. Results

### 3.1. Participants

General patient data can be found in Table 1. In total, the structural and functional MRI data sets, DSA data sets and neurological assessments of 69 patients were included in the study. Eight patients had prior revascularization surgery; consequently, 130 ICA territories and 260 ACA/MCA territories were evaluated with regard to the first aim of the study, in which the PIRAMID score was evaluated for its prognostic value regarding neurological symptomatology. The corresponding [^15^O]water PET data sets were available in 38 patients, of whom six patients had prior unilateral revascularization surgery. Accordingly, 140 ACA/MCA territories were evaluated in the second part of the study, in which the MARS-MMA scoring system was developed and compared to the PIRAMID scoring system for its capability to predict [^15^O]water PET impairment.

### 3.2. Assessment of the Components of the PIRAMID/MARS-MMA Scoring System, Neurological Symptomatology and [^15^O]Water PET

The frequencies of all components of the PIRAMID/MARS-MMA scoring systems (prior infarction on FLAIR/T2-weighted MRI, CVR impairment on bh-fMRI, arterial vessel wall contrast enhancement, Ivy sign, angiographic mSS and collateralization grading) and of neurological symptomatology and CPR impairment on [^15^O]water PET can be seen in Table 2.

The visual inspection of the cerebellar signal time-courses showed the accurate performance of the bh-fMRI tasks in all patients, so no bh-fMRI data set had to be excluded from further analysis.

The preliminary analysis to determine a bh-fMRI threshold value for further analysis revealed increasing sensitivity and decreasing specificity in predicting a severe CPR impairment with a rising CVR impairment (see Figure S1). A CVR impairment of 50% yielded the maximum Youden index, in both the analysis of the ICA territories (1.541) and the analysis of the ACA/MCA territories (1.567) (see Table S1). Therefore, a CVR impairment of ≥50% was used as a threshold to binarize the CVR.

The preliminary analysis of the arterial wall contrast enhancement showed that a mild arterial wall contrast enhancement yielded the maximum Youden index of 1.302 (see Figure S2). A mild arterial wall contrast enhancement was therefore used as a threshold to binarize the CVR impairment in the further analysis.

### 3.3. PIRAMID Scoring System

**Figure 2:**
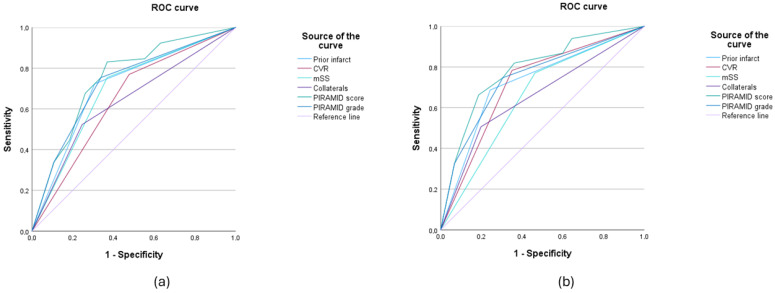
ROC curves of the PIRAMID components, the PIRAMID score and its grade with regard to the prediction of neurological symptomatology. In the evaluation of the territories of the internal carotid arteries (**a**), the AUCs of the PIRAMID score (0.749 *) and grade (0.724 *) were higher than the AUCs of all individual parameters (modified Suzuki Score (mSS) (0.692 *), impaired collateralization (0.638 *), prior infarcts (0.708 *), cerebrovascular reactivity (CVR) (0.646 *)). This was also evident in the evaluation of the territories of the anterior and the middle cerebral arteries (**b**): PIRAMID score (0.784 *), PIRAMID grade (0.745 *), mSS (0.654 *), impaired collateralization (0.654 *), prior infarcts (0.722 *), CVR (0.716 *)). * Confidence intervals not given since multiple territories (2 or 4) per patient were evaluated.

The frequencies of the calculated PIRAMID scores and grades are shown in Table 2. The ROC curve analysis of the ICA territories analogous to Ladner et al., with the neurological symptomatology as the dependent variable, yielded an AUC of 0.749 for the PIRAMID score and an AUC for the PIRAMID grade of 0.724 (see Figure 2a). Both the AUC of the PIRAMID score and that of the PIRAMID grade were higher than the AUCs of all PIRAMID components (see Figure 2a).

The evaluation of the MCA/ACA territories revealed an AUC of 0.784 for the PIRAMID score and an AUC of 0.745 for the PIRAMID grade (see Figure 2b). The AUC of the PIRAMID score and the AUC of the PIRAMID grade were higher than the AUCs of the PIRAMID components (see Figure 2b).

### 3.4. MARS-MMA Scoring System

A model with the variables CVR impairment, arterial vessel wall contrast enhancement and positive Ivy sign yielded a significant prediction of a severe [^15^O]water PET CPR impairment (see Table 3). Based on the optimized rounded weights for each factor (CVR impairment = 4, arterial vessel wall contrast enhancement = 3, Ivy sign = 2), a new scoring system was created (see Table 4). As the new scoring system consisted of only MRI-based components, we called it *MRI-Based Assessment of Risk of Stroke in Moyamoya Angiopathy* (MARS-MMA).

The MARS-MMA grade stratification with the following thresholds revealed the highest AUC (0.855): grade 1, 0 points; grade 2, 2–5 points; grade 3, 6–9 points (see Table 5 and Table S2).

### 3.5. Comparison of the PIRAMID Score and the MARS-MMA Score Regarding the Predictability of PET CPR Impairment on [^15^O]Water

**Figure 3:**
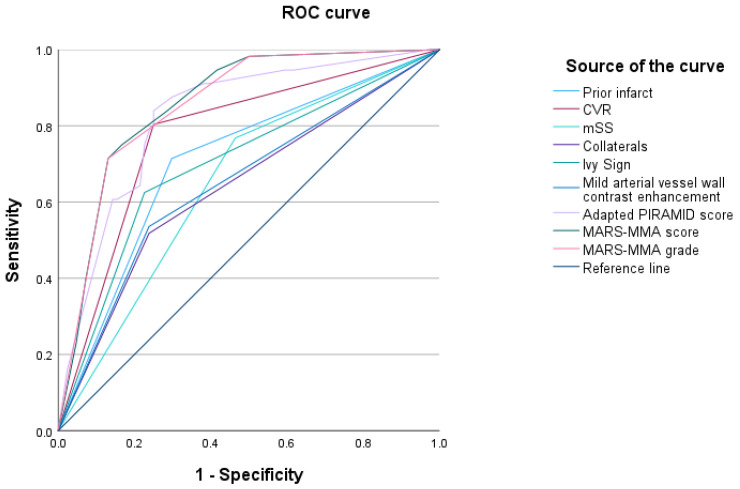
ROC curves of the MARS-MMA score, the MARS-MMA grade, the adapted PIRAMID score and all components of the scores predicting [^15^O]water PET cerebral perfusion reserve impairment. The AUC of the MARS-MMA score (0.859 *) was slightly higher than the AUC of the adapted PIRAMID score (0.827 *). The AUC of the MARS-MMA grade was 0.855 *. The AUCs of the components of the scores were lower (cerebrovascular reactivity (CVR) 0.777 *, modified Suzuki Score (mSS) 0.652 *, impaired collateralization 0.640 *, prior infarcts 0.708 *, arterial wall contrast enhancement 0.649 *, Ivy sign 0.699 *). * Confidence intervals not given since multiple territories (4) per patient were evaluated.

The weights of the adapted PIRAMID score can be seen in Table 6. The AUC of the MARS-MMA score (0.859) and the AUC of the MARS-MMA grade (0.855) were higher than the AUCs of all individual components of the score (see Figure 3). The comparison of the two scoring systems revealed a higher AUC for the MARS-MMA score than the adapted PIRAMID score (0.859 versus 0.827) (see Figure 3) and a lower AIC for the MARS-MMA score than the adapted PIRAMID score (140 versus 146).

### 3.6. Assessment of the MARS-MMA Grading Regarding the Prediction of CPR Impairment on [^15^O]Water PET

**Figure 4:**
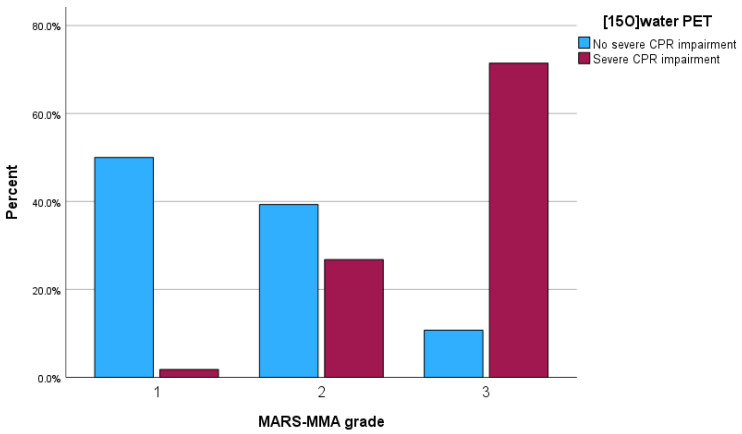
Bar graph showing MARS-MMA grade versus cerebral perfusion reserve (CPR) impairment on [^15^O]water PET. The negative predictive value impairment of MARS-MMA grade 1 was 97.7% and the positive predictive value of MARS-MMA grade 3 was 78.4%.

MARS-MMA grades 1–3 predicted a severe CPR impairment on [^15^O]water PET in 1/43 (2.3%) territories (MARS-MMA grade 1), 15/46% (32.6%) territories (MARS-MMA grade 2), and 40/51 (78.4%) territories (MARS-MMA grade 3) (see Figure 4). Consequently, the negative predictive value predicting the [^15^O]water PET CPR impairment of MARS-MMA grade 1 was 97.7% and the positive predictive value of MARS-MMA grade 3 was 78.4%. Nine of eleven territories with false-positive classification in MARS-MMA grade 3 yielded a reduced [^15^O]water PET CPR, albeit not below the arbitrary threshold of at least a severely reduced CPR (visual score ≥ 3) selected in the present study. A supplementary analysis based on the electronic medical records revealed, in nine of eleven of the territories, that surgical revascularization was indicated based on all clinical and imaging factors.

## 4. Discussion

In this study, we confirmed that the PIRAMID scoring system is helpful in predicting current or past neurological symptomatology. We evaluated not only the ICA territories, as proposed by Ladner et al., but also the ACA and MCA territories separately regarding past or current neurological symptoms and thereby obtained comparable results (AUC PIRAMID score 0.749 vs. 0.784, AUC PIRAMID grade 0.724 vs. 0.745). The AUC in our study was lower than in the study of Ladner et al. (PIRAMID score 0.749 vs. 0.860, PIRAMID grade 0.724 vs. 0.845).

Additionally, we intended to evaluated the PIRAMID scoring system with regard to the prediction of a severe CPR impairment on [^15^O]water PET. Certainly, patients’ past and current neurological symptomatology should be considered before the indication of neurosurgical revascularization. The aim of surgical intervention, however, is to prevent these ischemic events (ideally, in still asymptomatic patients), so the previous or current symptoms are not the best target values for the assessment of the risk of stroke. Additionally, silent strokes can be missed and will elude this evaluation. We therefore considered a CPR impairment on [^15^O]water PET to be the most important outcome, as it is already validated as a surrogate parameter for the risk of stroke [14].

Our second aim was to propose a new, entirely MRI-based classification system called MARS-MMA, which was optimized based on the [^15^O]water PET CPR impairment.

For statistical reasons, we calculated an adapted PIRAMID score (i.e., we optimized the weights of the predefined components of the PIRAMID score on our data) to avoid an unequal comparison between the MARS-MMA score, which was fitted to the current data, and the PIRAMID score. Both scoring systems revealed a strong ability to predict a severe CPR impairment, while the MARS-MMA scoring system predicted a CPR impairment slightly better than the PRIAMID scoring system (AUC 0.859 vs. 0.827), even after considering the inclusion of additional information (AIC = 140 vs. 146) in the model.

We determined three grades with low (MARS-MMA grade 1), intermediate (MARS-MMA grade 2), and high (MARS-MMA grade 3) risks of stroke. The analysis of the MARS-MMA grading revealed a strong ability to predict a severe CPR impairment (AUC 0.860), with high negative predictive value for MARS-MMA grade 1 (97.7%) and positive predictive value for MARS-MMA grade 3 (78.4%). A dedicated analysis of the territories with MARS-MMA grade 3 despite the “absence of a severely impaired” CPR indicated that, in 9 of 11 of the territories, surgical revascularization was indicated. This supports the usefulness of the MARS-MMA scoring system, which is intended to predict the risk of stroke and the indication for surgical revascularization. It should be considered that the four-point CPR rating and its subsequent binarization in this study were arbitrary and used for simplification, and this is not the gold standard for clinical decision-making. The results suggest that, for territories with MARS-MMA grade 1 and MARS-MMA grade 3, the reliable selection of patients with a very low and very high risk of stroke is possible with MR imaging alone. In territories with MARS-MMA grade 2 and an intermediate risk of stroke, we would recommend a [^15^O]water PET examination or early MRI examinations to reevaluate the MARS-MMA score and grade.

The MARS-MMA scoring system consists of the parameters of reduced CVR obtained from bh-fMRI, arterial vessel wall contrast enhancement and the presence of the Ivy sign on FLAIR images. Interestingly, the regression analysis showed no improvement in the prediction of CPR impairment with the additional evaluation of angiographic findings.

As the CVR is known to correlate highly with the CPR, it is not surprising that it became the main component of the scoring system. As opposed to Ladner et al. and van Niftrik et al., we applied bh-fMRI instead of hypercapnia-triggered fMRI with the use of the inhalation of CO_2_-enriched gas. This method requires no complex inhalation and monitoring equipment and therefore benefits from better availability [8,10,15]. Undoubtedly, one disadvantage is the need for patients’ cooperation and the ability to perform breath-hold tasks [8,10,15]. Still, this study indicates that bh-fMRI appears to be suitable for the MARS-MMA system. Furthermore, the validation of the PIRAMID scoring system suggests that bh-fMRI shows comparable results to CO_2_-inhalation-triggered fMRI when used with the PIRAMID score (AUC = 0.738 (ICA territories) and 0.771 (ACA/MCA territories) vs. 0.716) [17]. Another component of the MARS-MMA scoring system is the presence of the Ivy sign on FLAIR/T2-weighted images. The Ivy sign is considered an indicator of slow or retrograde flow in the cortical vessels [28]. After revascularization, the sign can diminish or temporally become more apparent [28]. Likewise, arterial vessel wall contrast enhancement is considered a sign of disease progression [7] and therefore represents a dynamic parameter likely ending in progredient stenosis with CPR/CVR impairment.

One major advantage of the MARS-MMA scoring system over the PIRAMID scoring system is that it is entirely MRI-based. The MARS-MMA scoring system requires no invasive DSA examination and is not accompanied by radiation exposure. For this reason, the evaluation of the MARS-MMA score can be repeated as often as desired based on patients’ therapy and symptoms. All components of the MARS-MMA score can be acquired in the same MR imaging session. Another advantage of the MARS-MMA scoring system is that it predicts the risk of reduced CPR and indirectly the risk of stroke in the vascular territories of the ACA and the MCA, and not only of the entire ICA territories. In our opinion, this is important as, at our center and other moyamoya centers, not only the MCA territories but also the ACA (and PCA) territories are revascularized if required. Therefore, the evaluation of the risk of stroke for all individual vascular territories is of high clinical relevance. However, the PCA territories were excluded in this analysis as their involvement and need for revascularization is only seen in less than 10% of cases. Since, in this study, we found that the PIRAMID score provided comparable accuracy in the neurological assessment of the ACA/MCA territories and ICA territories, we recommend the more detailed ACA/MCA evaluation for future use.

**Figure 5:**
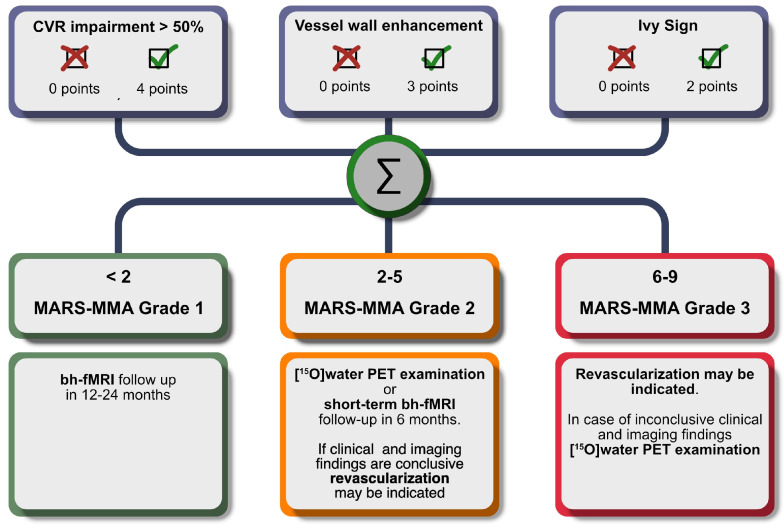
Flowchart of MARS-MMA score calculation and recommendations for therapeutic and diagnostic procedures for each of the 3 grades. Note: All suggested clinical decisions must be correlated with the clinical findings for final decision-making in each case.

The aim of the MARS-MMA score and especially of the MARS-MMA grading is to simplify clinical diagnostics by pre-selecting patients for future treatment decisions (see Figure 5).

MARS-MMA grade 1: At this stage of the disease, there is likely only mild perfusion restriction with a near to normal cerebrovascular reserve capacity and only little or no risk of short-term deterioration. We recommend follow-up with breath-hold fMRI and the re-evaluation of the MARS-MMA score in 12–24 months.

MARS-MMA grade 2: The impairment of the cerebrovascular reserve capacity and a decline in the near future are possible. Depending on the clinical presentation, we recommend an additional [^15^O]water PET scan or a short-term bh-fMRI follow-up in 6 months. If the clinical presentation and the imaging findings are conclusive, revascularization surgery may be indicated. 

MARS-MMA grade 3: MARS-MMA grade 3 is suggestive of severe CVR impairment with a high risk of subsequent stroke. Revascularization may be indicated. In the case of inconclusive clinical and/or imaging findings, timely [^15^O]water PET scanning is recommended to assess the need for revascularization.

With this approach, we aim to avoid unnecessary MRI and [^15^O]water PET examinations and enable the best possible treatment for patients. One limitation of this study is the limited sample size and its retrospective nature. In addition, we used the CPR impairment on [^15^O]water PET as a surrogate parameter for the risk of stroke, instead of assessing the factual occurrence of strokes. It is important to emphasize that we pragmatically rated the CPR on [^15^O]water PET using a 4-point score and arbitrarily decided to binarized each ACA/MCA territory regarding severe CPR impairment (visual CPR score of ≥3) or a normal/not severely impaired CPR (visual CPR score < 3). This binarization was performed to provide comparability to the statistics of the study of Ladner et al. However, a stepwise linear regression analysis with a 4-point-scored CPR as an independent variable (without binarization) yielded similar results. The aim of this evaluation was to show the strong correlation between the MARS-MMA score and the CPR. The MARS-MMA grades currently generated from the MARS-MMA scores should therefore be seen as a preliminary suggestion for further prospective studies. Another limitation is that, due to the limited sample size, we were unable to validate the MARS-MMA score in an independent control cohort to avoid overfitting. Future studies should validate the results in a larger number of patients and evaluate the extent to which the score estimates the true risk of stroke by prospectively comparing the initially attributed score and grade with the occurrence of future TIA and strokes.

## 5. Conclusions

In this study, we outlined the new, entirely non-invasive MRI-based scoring system MARS-MMA to predict the risk of stroke in moyamoya angiopathy. The components of the MARS-MMA scoring system include the evidence of a reduced CVR with the use of bh-fMRI, the presence of an arterial vessel wall contrast enhancement and the presence of the Ivy sign on FLAIR images. The MARS-MMA scoring system correlates well with a reduced CPR on [^15^O]water PET and therefore might be helpful in clinical decision-making. A further prospective evaluation is mandatory to validate its predictive value in a larger cohort.

## Figures and Tables

**Table 1:** General patient data.

Mean age (range)	46 (15–75)
Female–male ratio	54:17
Imaging before revascularization	61
Imaging after unilateral revascularization	8
Structural MRI data sets	69
Functional MRI data sets	69
DSA data sets	69
[^15^O]water PET data sets	38
Days between MRI and DSA data acquisition (mean ± SD)	11 ± 25
Days between MRI and [^15^O]water PET data acquisition (mean ± SD)	50 ± 36
Days between DSA and [^15^O]water PET data acquisition (mean ± SD)	53 ± 34

**Table 2:** Clinical data.

	Analysis of the Territories of the Internal Carotid Artery	Analysis of the Territories of the Anterior and Middle Cerebral Arteries
Neurological symptomatology	65/130 (50.0%)	83/260 (31.9%)
Motor deficit ^1^	30/130 (23.1%)	58/260 (22.3%)
Sensory deficit ^1^	59/130 (45.4%)	79/260 (30.4%)
Language deficit ^1^	24/64 (37.5%) ^2^	24/64 (37.5%) ^2^
Prior infarct	67/130 (51.5%)	100/260 (38.5%)
Cerebrovascular reactivity impairment		
≥10% relative BOLD signal reduction	115/130 (88.5%)	213/260 (81.9%)
≥20% relative BOLD signal reduction	110/130 (84.6%)	191/260 (73.5%)
≥30% relative BOLD signal reduction	104/130 (80.0%)	178/260 (68.5%)
≥40% relative BOLD signal reduction	94/130 (72.3%)	158/260 (60.8%)
≥50% relative BOLD signal reduction	81/130 (62.3%)	127/260 (48.8%)
≥60% relative BOLD signal reduction	64/130 (49.2%)	92/260 (35.4%)
≥70% relative BOLD signal reduction	58/130 (44.6%)	76/260 (29.2%)
≥80% relative BOLD signal reduction	47/130 (36.2%)	56/260 (21.5%)
≥90% relative BOLD signal reduction	44/130 (33.8%)	49/260 (18.8%)
100% relative BOLD signal reduction	40/130 (30.8%)	43/260 (16.5%)
Steal Phenomenon	36/130 (27.7%)	36/260 (13.8%)
Arterial wall contrast enhancement		
Mild	-	50/140 (35.7%)
Moderate	-	32/140 (22.9%)
Strong	-	25/140 (17.9%)
Presence of Ivy sign	-	54/140 (38.6%)
Modified Suzuki Score ≥ II	73/130 (56.2%)	146/260 (56.2%)
Impaired collateralization in ≥2 territories	50/130 (38.5%)	77/260 (29.6%)
Indication for neurosurgical revascularization	-	65/140 (46.4%)
PIRAMID score		
0	29/130 (22.3%)	68/260 (26.2%)
1	10/130 (7.7%)	14/260 (5.4%)
3	13/130 (10.0%)	46/260 (17.7%)
4	7/130 (5.4%)	16/260 (6.2%)
6	10/130 (7.7%)	28/260 (10.8%)
7	20/130 (15.4%)	32/260 (12.3%)
9	12/130 (9.2%)	17/260 (6.5%)
10	29/130 (22.3%)	39/260 (15.0%)
PIRAMID grade		
1	59/130 (45.4%)	144/260 (55.4%)
2	42/130 (32.3%)	77/260 (29.6%)
3	29/130 (22.3%)	39/260 (15.0%)
Severe [^15^O]water PET cerebral perfusion reserve impairment	36/70 (51.4%)	55/140 (39.3%)

^1^ Combinations of symptoms possible; ^2^ 64 evaluated territories of the left middle cerebral artery and the left internal carotid artery supplying the language-dominant hemisphere in right-handed individuals.

**Table 3:** Results of the forward and backward binary logistic regression analysis with [^15^O]water PET cerebral perfusion reserve impairment as the dependent variable.

Variable	Odds Ratio	*p* Value	95% Confidence Interval	Regression Coefficient
bh-fMRI CVR impairment > 50%	9.300	<0.001	3.702–23.361	2.230
Arterial vessel wall contrast enhancement	4.382	0.002	1.752–10.961	1.477
Ivy sign	3.167	0.012	1.291–7.766	1.153

Variables not included in the model: mSS (*p* = 0.176), collaterals (*p* = 0.565), prior infarct (*p* = 0.164).

**Table 4:** The MARS-MMA scoring system.

Variable	Points
Breath-hold fMRI cerebrovascular reactivity impairment (CVR) of at least 50% compared to the cerebellar CVR response	4
Arterial vessel wall contrast enhancement	3
Presence of Ivy sign on FLAIR images	2

**Table 5:** The MARS-MMA grades.

MARS-MMA Grade	MARS-MMA Points
1	0
2	2–5
3	6–9

**Table 6:** Results of the binary logistic regression analysis with [^15^O]water PET cerebral perfusion reserve impairment as the dependent variable and block entry of the independent variables.

Variable	Odds Ratio	*p* Value	95% Confidence Interval	Regression Coefficient
mSS	1.655	0.312	0.628–4.397	0.504
Collaterals	0.913	0.856	0.342–2.435	−0.091
Prior infarct	2.812	0.022	1.160–6.818	1.034
bh-fMRI CVR impairment > 50%	7.289	<0.001	2.863–18.603	1.988

## Data Availability

In order to safeguard the confidentiality of the participants, the data pertaining to this study are currently withheld from public access. The data can be shared upon request.

## Supplementary Materials

The following supporting information can be downloaded at: https://www.mdpi.com/article/10.3390/diagnostics14131437/s1, Figure S1: Preliminary analysis of the extent to which breath-hold fMRI (bh-fMRI) cerebrovascular reactivity (CVR) impairment best predicts [^15^O]water PET cerebral perfusion reserve impairment; Figure S2: Preliminary analysis of the extent to which arterial wall contrast enhancement best predicts CPR impairment on [^15^O]water PET: Mild arterial wall contrast enhancement; Table S1: Area under the curve for breath-hold fMRI (bh-fMRI) cerebrovascular reactivity (CVR) predicting [^15^O]water PET cerebral perfusion reserve impairment; Table S2: All possible 3-point grade stratifications achievable through the combination of the weighted factors of the MARS-MMA score and the corresponding area under the curve indicating [^15^O]water PET cerebral perfusion reserve impairment.
