# Supplementary material for: MRI-Based Assessment of Risk for Stroke in Moyamoya Angiopathy (MARS-MMA): An MRI-Based Scoring System for the Severity of Moyamoya Angiopathy
Source: Diagnostics (Basel). 2024 Jul 5;14(13):1437. doi: 10.3390/diagnostics14131437 (PMC11241620; doi:10.3390/diagnostics14131437)
Supplement: Supplementary file 1 [file diagnostics-14-01437-s001.zip › TableS1.pdf]

Table S1. Area under the curve for breath-hold fMRI (bh-fMRI) cerebrovascular reactivity (CVR) predicting [^{15}O]water PET cerebral perfusion reserve impairment

bh-fMRI CVR impairment compared to the cerebellar CVR response	Territories of the internal carotid artery Youden index	Territories of the anterior and middle cerebral artery Youden index
0 %	1.000	1.000
10 %	1.282	1.176
20 %	1.315	1.235
30 %	1.350	1.297
40 %	1.460	1.389
50 %	1.541	1.567
60 %	1.376	1.432
70 %	1.302	1.407
80 %	1.329	1.353
90 %	1.317	1.382
100 %	1.274	1.356
Steal phenomenon	1.231	1.358
