# Supplementary material for: MRI-Based Assessment of Risk for Stroke in Moyamoya Angiopathy (MARS-MMA): An MRI-Based Scoring System for the Severity of Moyamoya Angiopathy
Source: Diagnostics (Basel). 2024 Jul 5;14(13):1437. doi: 10.3390/diagnostics14131437 (PMC11241620; doi:10.3390/diagnostics14131437)
Supplement: Supplementary file 1 [file diagnostics-14-01437-s001.zip › TableS2.pdf]

Table S2. All possible 3-point grade stratifications achievable by combination of the weighted factors of the MARS-MMA score and corresponding area under the curve indicating [^{15}O]water PET cerebral perfusion reserve impairment

Grade 1	Grade 2	Grade 3	Area under the curve
0	2	3 - 9	0.773
0	2 - 3	4 - 9	0.811
0	2 - 4	5 - 9	0.847
0	2 - 5	6 - 9	0.855
0	2 - 6	7 - 9	0.804
0	2 - 7	9	0.776
0 - 2	3	4 - 9	0.805
0 - 2	3 - 4	5 - 9	0.847
0 - 2	3 - 5	6 - 9	0.852
0 - 2	3 - 6	7 - 9	0.813
0 - 2	3 - 7	9	0.791
0 - 3	4	5 - 9	0.814
0 - 3	4 - 5	6 - 9	0.824
0 - 3	4 - 6	7 - 9	0.803
0 - 3	4 - 7	9	0.790
0 - 4	5	6 - 9	0.802
0 - 4	5 - 6	7 - 9	0.798
0 - 4	5 - 7	9	0.793
0 - 5	6	7 - 9	0.792
0 - 5	6 - 7	9	0.790
0 - 6	7	9	0.660
