# Supplementary figures and images for: MRI-Based Assessment of Risk for Stroke in Moyamoya Angiopathy (MARS-MMA): An MRI-Based Scoring System for the Severity of Moyamoya Angiopathy
Source: Diagnostics (Basel). 2024 Jul 5;14(13):1437. doi: 10.3390/diagnostics14131437 (PMC11241620; doi:10.3390/diagnostics14131437)

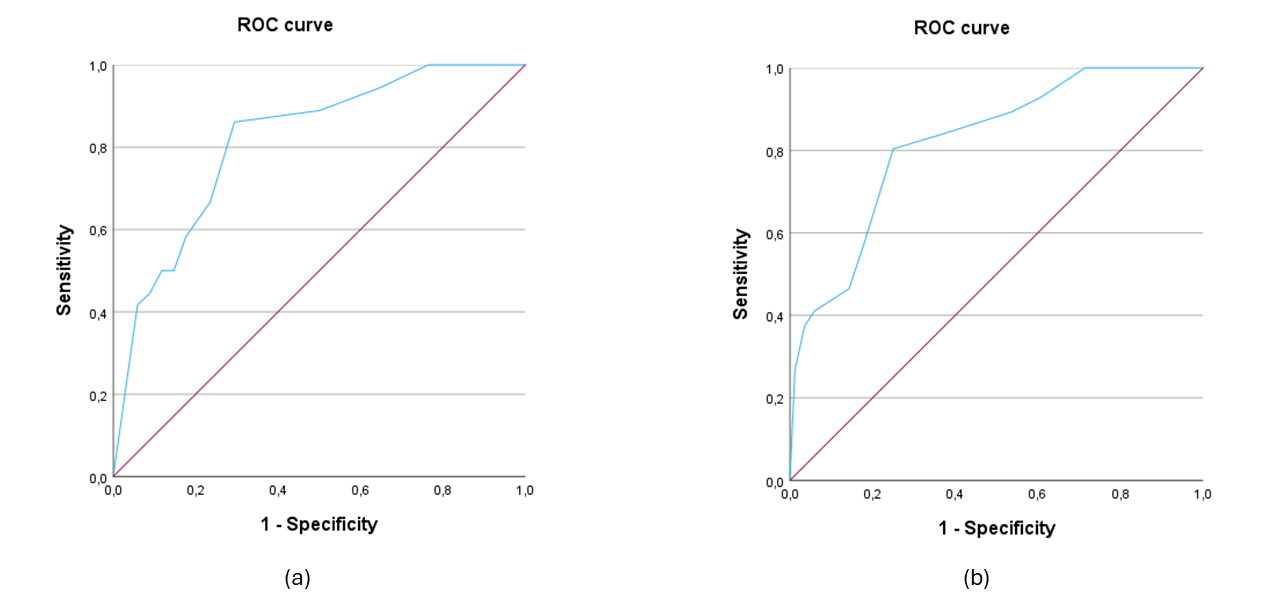

Supplement: Supplementary file 1 [file diagnostics-14-01437-s001.zip › FigS1.tif]

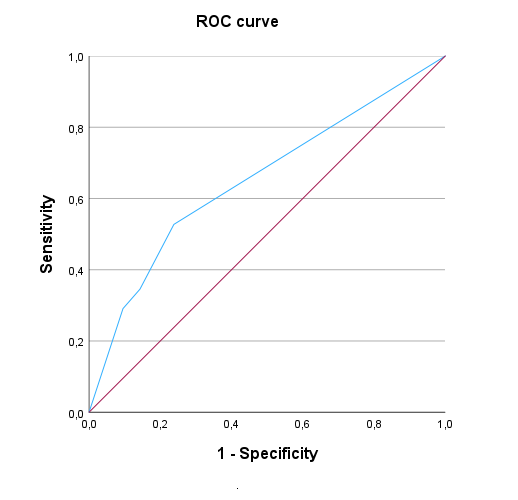

Supplement: Supplementary file 1 [file diagnostics-14-01437-s001.zip › FigS2.png]
